# Prediction of angiographic coronary disease and mortality with a cadmium-zinc-telluride camera: a comparison of upright and supine ejection fractions and left ventricular volumes

**DOI:** 10.3389/fnume.2023.1162784

**Published:** 2023-04-28

**Authors:** Jackson Walker, Annette Christianson, Muhammad Athar, Fahad Waqar, Myron Gerson

**Affiliations:** ^1^ Department of Internal Medicine, University of Cincinnati College of Medicine, Cincinnati, OH, United States; ^2^ Division of Statistics and Data Science, University of Cincinnati College of Medicine, Cincinnati, OH, United States; ^3^ Cardiopulmonary Department, Adena Regional Medical Center, Chillicothe, OH, United States; ^4^ Division of Cardiovascular Health and Disease, Department of Internal Medicine, University of Cincinnati College of Medicine, Cincinnati, OH, United States

**Keywords:** CZT SPECT, D-SPECT, upright SPECT, supine SPECT, LVEF, ESV

## Abstract

**Introduction:**

Perfusion imaging strongly predicts coronary artery disease (CAD), whereas cardiac volumes and left ventricular ejection fraction (LVEF) strongly predict mortality. Compared to conventional Anger single-photon emission computed tomography (SPECT) cameras, cadmium-zinc-telluride (CZT) cameras provide higher resolution, resulting in different left ventricular volumes. The cadmium-zinc-telluride D-SPECT camera is commonly used to image in the upright position, which introduces changes in left ventricular loading conditions and potentially alters left ventricular volumes. However, little or no data exist on the predictive value of left ventricular volumes and ejection fraction when acquired in the upright position. We investigated models for the prediction of CAD and mortality, comparing upright and supine imaging.

**Methods:**

A retrospective study of patients with upright/supine stress and rest imaging and coronary angiography within 3 months was performed. Univariate and multivariable analyses were performed to predict abnormal angiograms and all-cause mortality.

**Results:**

Of the 392 patients, 210 (53.6%) had significant angiographic CAD; 78 (19.9%) patients died over 75 months. The best multivariable model for CAD included the supine summed stress score and supine stress LVEF, with an area under the receiver operating characteristic of 0.862, a sensitivity of 76.7%, and a specificity of 82.4%, but this model was not statistically superior to the best upright model. The best multivariable models for mortality included age, diabetes, history of cardiovascular disease, and end-systolic volume, with the upright and supine models being equivalent.

**Discussion:**

Angiographic CAD was best predicted by the supine summed stress score and LVEF but was not statistically superior to the next-best upright model. Mortality was best predicted by end-systolic volume in combination with age, diabetes status, and cardiovascular disease status, with equivalent results from the upright and supine images.

## 1. Introduction

The advent of digital cadmium-zinc-telluride (CZT) single-photon emission computed tomography (SPECT) imaging ushered in an era of faster, more patient-friendly perfusion stress testing (1, 2). With these new devices came the need to validate and reconcile the effects of increased spatial resolution on attenuation artifacts and volumetric measurements (3–5). With respect to clinical outcomes, the resulting CZT SPECT literature has focused primarily on myocardial perfusion findings for the prediction of coronary artery disease (CAD), cardiac events, and mortality (6–14). Although left ventricular ejection fraction (LVEF) and volumes are known to be strong predictors of mortality, the prognostic value of CZT SPECT volumetric measurements has been less explored (15–22). In a multicenter comparative study of the Anger camera and CZT camera measurements of LVEF, the mean LVEF was found to be significantly higher with the CZT camera (23). The prognostic implications of this difference in LVEF are unknown.

An important factor affecting CZT SPECT volumetric measurements is patient positioning. Unlike other imaging modalities obtained in a single supine position, CZT SPECT studies are commonly performed in two positions to reduce the risk of incorrectly interpreting attenuation artifacts as perfusion abnormalities (24–28). This potentially provides the imager with both upright and supine LVEF and volume data. No study, however, has directly compared the relative prognostic power of LVEF vs. left ventricular volumes on a CZT camera, and no study has addressed whether the prognostic implications are different in the upright vs. supine position.

There is limited previous research on the accuracy of LVEF and volume measurements obtained in the upright position (24, 29–31). Previous data in healthy participants (30, 32) and patients referred for evaluation of angina pectoris (24) have shown that resting end-diastolic volumes (EDV) and end-systolic volumes (ESV) are generally smaller in the upright position compared to the supine position, whereas LVEF is more variable. It is unclear whether these differences may affect the prognostic value of upright volumetric measurements on a CZT SPECT camera.

The aim of this study was to compare the prognostic value of upright vs. supine volumetric imaging for the prediction of CAD and mortality by creating multivariable prediction models. Because left ventricular volumes and ejection fractions differ due to differences in ventricular loading, we hypothesized that there may be a difference in the prediction of mortality between the upright and supine images acquired with a CZT camera.

## 2. Materials and methods

### 2.1. Study population and clinical data collection

All methods for this retrospective cohort study were reviewed and approved by the Institutional Review Board of the University of Cincinnati, with an exception for informed consent. The study population underwent upright and supine SPECT myocardial perfusion imaging with a Spectrum Dynamics D-SPECT camera (Spectrum Dynamics Inc., Palo Alto, CA, United States) between 20 June 2014 and 4 February 2016. All patients who underwent selective coronary angiography within 3 months of perfusion imaging were included in the present study, regardless of other medical histories.

Clinical data were obtained from the electronic medical record (Epic Systems, Verona, WI, USA). All-cause mortality data were acquired from the local medical record and from communicating institutions utilizing the same electronic medical record. Follow-up was terminated at death or last contact with the electronic medical record.

### 2.2. SPECT protocol

Our SPECT protocol was conducted as follows. Patients were counseled to avoid caffeinated beverages 24 h before testing and to avoid beta-blockers or calcium channel antagonists 24–48 h before testing, unless otherwise requested by the referring physician. Exercise testing was performed on a treadmill using the Bruce protocol. Pharmacologic stress was performed with regadeoson 0.4 mg intravenously when exercise testing was not possible.

Upright and supine stress and rest images were acquired with a D-SPECT CZT dedicated cardiac camera. Upright imaging was performed with the imaging chair at an angle of 65°–70°. Rest images were acquired for 3–11 min at 60 min following a weight-based injection of 9.4–14 mCi (mean 0.11 mCi/kg) of technetium-99 m tetrofosmin. Stress images were acquired for at least 3 min at 30–45 min following a weight-based injection of 27.7–42 mCi (mean 0.33 mCi/kg) of technetium-99 m tetrofosmin.

### 2.3. Image processing

Images were processed using iterative reconstruction (ordered subset expectation maximization) on a Spectrum Dynamics Cedars View processing station (Spectrum Dynamics Inc., Palo Alto, CA, United States). Processed images were reviewed by a physician reader and inaccurate computer identification of endocardial borders, the left ventricular long axis, and apical or basal planes was corrected at the time of image interpretation. The interpretation was performed by two experienced nuclear cardiologists using Corridor4DM SPECT software (INVIA Medical Imaging Solutions, Ann Arbor, MI, United States). Differences in interpretation were resolved by consensus agreement. The studies were read independently, without knowledge of the test indication or clinical data other than gender, height, and weight. Summed stress score (SSS), summed rest score, and summed difference score were recorded from the upright and supine positions. Upright and supine rest and post-stress LVEF, ESV, and EDV were calculated from the gated images by the 4DM SPECT software.

### 2.4. Method for CAD assessment

Coronary angiographic images were blinded for the patient's age, gender, past medical history, presenting symptoms, and SPECT results. Angiographic CAD was defined as a stenosis of ≥50% in the left main coronary artery and/or ≥70% in the left anterior descending, left circumflex, right coronary artery, or main branch. Angiographic CAD was assessed by an independent, blinded interventional cardiologist. This assessment was compared to the documented reading in the patient's medical chart, and any differences were resolved by a second blinded interventional cardiologist.

### 2.5. Statistical methods

Categorical variables were summarized using frequencies and percentages, with chi-squared tests used for comparisons. Continuous variables were summarized using means and standard deviations, and *t*-tests were used for comparisons. Logistic regression models were used to predict angiographic CAD, and Cox regression models were used to predict mortality. For each outcome, demographic and clinical variables were evaluated in univariate models. Multivariable models were then constructed using forward stepwise selection, using demographic variables only, demographic variables plus upright stress results, demographic variables plus upright rest results, demographic variables plus supine stress results, and demographic variables plus supine rest results. The best logistic and Cox models were chosen using the C-statistic and Akaike information criterion (AIC), respectively; models were compared using Vuong's closeness test and partial likelihood ratio test, respectively. All statistical analyses were performed with SAS version 9.4. Figures were generated using SAS 9.4 software.

## 3. Results

### 3.1. Demographic results

Between 20 June 2014 and 4 February 2016, 2,779 patients underwent SPECT testing in the Nuclear Cardiology Laboratory. Of these patients, 395 underwent both upright and supine rest and stress imaging and subsequently underwent selective coronary angiography within 3 months and were included in the present study. Three patients were excluded from the final analysis due to missing perfusion data. Of the 392 patients, 169 (43.1%) were women; 98 (25.0%) patients underwent exercise stress, and the remaining patients underwent pharmacological stress. Of the 392 patients, 210 (53.6%) had significant angiographic CAD, and 78 (19.9%) died during a median follow-up of 75 months. See Table 1 for complete patient demographic data.

**Table 1:** Study population demographics and outcome frequencies.

	Total (*n* = 392)	Survivors (*n* = 314, 80.1%)	Non-survivors (*n* = 78, 19.9%)	*p*-value
Age (mean, SD)	58.9 (10.4)	62.9 (9.5)	57.9 (10.4)	0.0001*
Gender (*n*, %)				0.0139*
Men	223 (56.9)	169 (53.8)	54 (69.2)	
Women	169 (43.1)	145 (46.2)	24 (30.8)	
BMI (mean, SD)	32.6 (8.1)	30.6 (6.7)	33.1 (8.4)	0.0044*
BSA (mean, SD)	2.1 (0.3)	2.1 (0.3)	2.1 (0.3)	0.4082
Pharm/exercise test (*n*, %)				0.3065
Pharm	294 (75.0)	232 (73.9)	62 (79.5)	
Exercise	98 (25.0)	82 (26.1)	16 (20.5)	
Diabetes(*n*,%)	156 (39.8)	119 (37.9)	37 (47.4)	0.1235
Hypertension (*n*, %)	314 (80.1)	247 (78.7)	67 (85.9)	0.1520
HLD (*n*, %)	188 (48.0)	143 (45.5)	45 (57.7)	0.0545
CAD (*n*, %)	98 (25.0)	65 (20.7)	33 (42.3)	<0.0001*
CVD (*n*, %)	148 (37.8)	103 (32.8)	45 (57.7)	<0.0001*
CMP (*n*, %)	77 (19.6)	50 (15.9)	27 (34.6)	0.0002*
Angiographic CAD (*n*, %)	210 (53.6)	154 (49.0)	56 (71.8)	0.0003*

SD, standard deviation, BMI, body mass index; BSA, body surface area; HLD, hyperlipidemia; CAD, history of coronary artery disease; CVD, cardiovascular disease, defined as previously; CAD, cerebrovascular disease and/or peripheral arterial disease; CMP, cardiomyopathy, defined as a history of reduced ejection fraction and/or heart failure.

* Significant *p-*values < 0.05.

### 3.2. Perfusion scores and left ventricular volumes

Perfusion scores and volumetric data are presented in Table 2. The mean differences in perfusion scores between the upright and supine positions were not statistically significant. Stress and rest LVEF and stress and rest EDV were all lower in the upright position than in the supine position, but the absolute differences were small. The differences between upright and supine ESV at rest (0.3 ml) and stress (0.3 ml) were minimal and not statistically different.

**Table 2:** Upright vs. supine perfusion and volumetric variables.

	Upright (mean, SD)	Supine (mean, SD)	*p*-value, upright vs. supine
SSS	4.4 (5.7)	4.6 (5.6)	0.1971
SRS	2.7 (4.0)	2.9 (4.4)	0.2096
SDS	2.3 (2.9)	2.3 (2.8)	0.9691
Stress LVEF	51.9 (13.9)	52.9 (13.6)	0.0006*
Rest LVEF	52.2 (14.1)	53.3 (13.7)	0.0015*
Difference stress vs. rest LVEF	0.0 (0.1)	0.0 (0.1)	0.968
Stress ESV	68.8 (50.2)	68.5 (48.4)	0.5074
Rest ESV	65.5 (46.8)	65.2 (44.6)	0.5653
Difference stress vs. rest ESV	3.3 (10.9)	3.3 (12.1)	0.9869
Stress EDV	130.4 (57.3)	133.5 (55.9)	<0.0001*
Rest EDV	125.4 (53.9)	128.6 (51.5)	<0.0001*
Difference stress vs. rest EDV	5.0 (13.5)	4.9 (15.1)	0.9261

SRS, summed rest score; SDS, summed difference score; SSS, summed stress score; LVEF, left ventricular ejection fraction; EDV, end-diastolic volume; ESV, end-systolic volume.

* Significant *p*-values < 0.05.

### 3.3. Univariate analysis for CAD prediction

Table 3 shows the univariate analysis for CAD. The strongest upright predictors for CAD were SSS [area under the receiver operating characteristic (AUROC) 0.823], summed difference score (AUROC 0.778), summed rest score (AUROC 0.709), and stress LVEF (AUROC 0.706). The strongest supine predictors were SSS (AUROC 0.848), summed difference score (AUROC 0.781), summed rest score (AUROC 0.738), and stress LVEF (AUROC 0.682). Supine SSS was not independently statistically superior to upright SSS for the prediction of CAD. The presence of hyperlipidemia (AUROC 0.568, *p* = 0.0073), known cardiovascular disease (AUROC 0.570, *p* = 0.0044), cardiomyopathy (AUROC 0.581, *p* < 0.0001), and a pharmacological stress type (AUROC 0.544, *p* = 0.0477) were all significantly but weakly predictive of CAD.

**Table 3:** Individual SPECT variables AUROC for prediction of CAD.

	Upright		Supine	
	AUROC	*p*-value	AUROC	*p*-value
SSS	0.823	<0.0001*	0.848	<0.0001*
SRS	0.709	<0.0001*	0.738	<0.0001*
SDS	0.778	<0.0001*	0.781	<0.0001*
Stress LVEF	0.706	<0.0001*	0.682	<0.0001*
Rest LVEF	0.643	<0.0001*	0.662	<0.0001*
Stress EDV	0.646	<0.0001*	0.65	<0.0001*
Rest EDV	0.645	<0.0001*	0.633	<0.0001*
Stress ESV	0.679	<0.0001*	0.671	<0.0001*
Rest ESV	0.648	<0.0001*	0.654	<0.0001*
Difference stress LVEF − rest LVEF^a^	0.585	0.0017*	0.537	0.2946
Difference stress EDV − rest EDV^a^	0.569	0.0083*	0.611	0.0004*
Difference stress ESV − rest ESV^a^	0.634	<0.0001*	0.622	0.0003*

SPECT, single-photon emission computed tomography; AUROC, area under the receiver operating characteristic; CAD, coronary artery disease; SRS, summed rest score; SDS, summed difference score; SSS, summed stress score; LVEF, left ventricular ejection fraction; EDV, end-diastolic volume; ESV, end-systolic volume.

* Significant *p*-values < 0.05.

^a^ Difference values refer to the absolute difference between stress and rest measurements.

### 3.4. Multivariable analysis for CAD prediction

**Figure 1:**
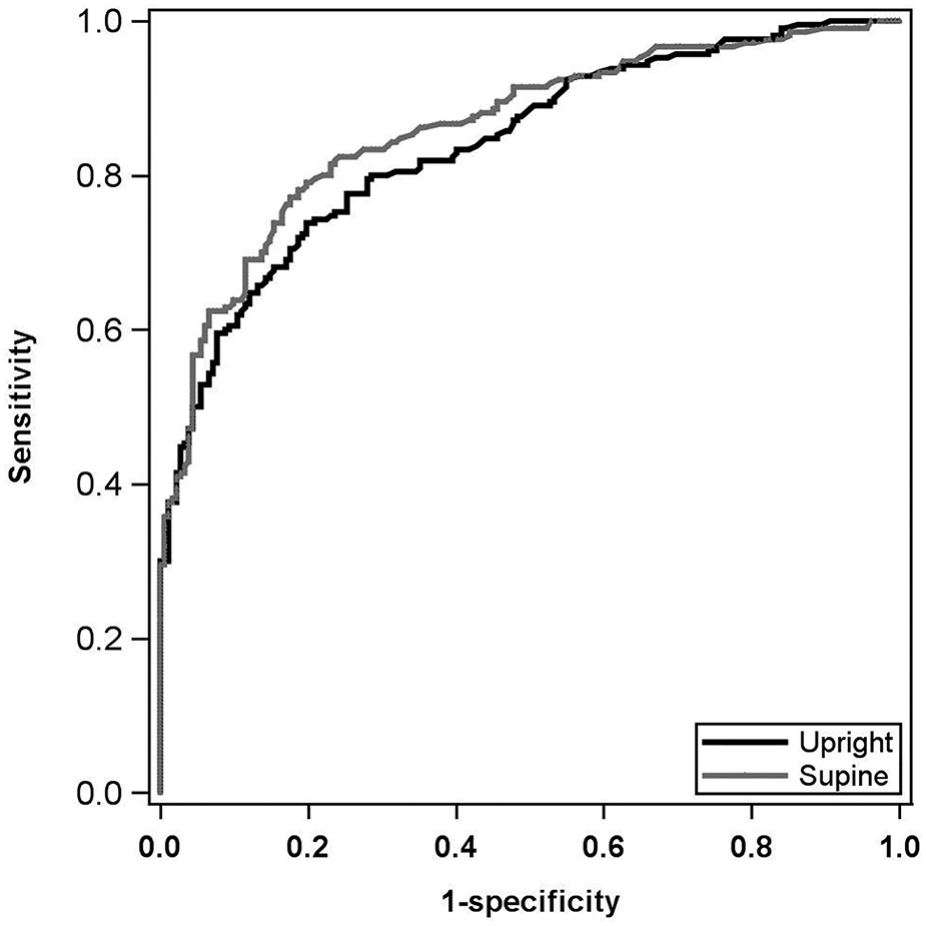
Multivariable CAD AUROC curves. Black curve: best upright CAD prediction model. Includes upright SSS, upright stress LVEF, and HLD status. AUROC: 0.839; sensitivity: 70.5%; specificity: 81.9%. Gray curve: best supine CAD prediction model. Includes supine SSS and supine stress LVEF. AUROC: 0.862; sensitivity: 76.7%; specificity: 82.4%. Models were not statistically different by Vuong's closeness test. CAD, coronary artery disease; AUROC, area under the receiver operating characteristic; SSS, summed stress score; LVEF, left ventricular ejection fraction; HLD, hyperlipidemia.

Multivariable modeling for CAD prediction demonstrated that the supine SSS and supine stress LVEF model was the best predictor of CAD with an AUROC of 0.862, a sensitivity of 76.7%, and a specificity of 82.4%. The best upright model consisted of upright SSS, supine LVEF, and a history of hyperlipidemia with an AUROC of 0.839, a sensitivity of 70.5%, and a specificity of 81.9% (Figure 1). Vuong's closeness test was performed between these upright and supine models and showed no statistical difference.

### 3.5. Univariate analysis for mortality prediction

The univariate analysis for all-cause mortality is presented in Table 4. The strongest upright predictor of mortality was stress EDV, followed by stress ESV, rest ESV, and rest EDV in ascending order of AIC. The lower the AIC value, the better the predictive ability of the variable. The strongest supine predictor was stress ESV, followed by stress EDV, rest ESV, and rest LVEF in ascending order of AIC. The demographic variables that predicted mortality were increasing age, male gender, increasing body mass index (BMI), cardiovascular disease, and cardiomyopathy. The change in the left ventricular end-diastolic volume between the upright (mean 129.3 ml) and supine (mean 132.8 ml) positions in patients with a body mass index <35 kg/m^2^ was 3.5 ml. For a body mass index ≥35 kg/m^2^, the mean change in the left ventricular end-diastolic volume between the upright (mean 132.5 ml) and supine (mean 135.0 ml) positions was 2.5 ml (*p* = NS).

**Table 4:** Individual SPECT variables and AIC for prediction of all-cause mortality.

	Upright		AIC	Supine		AIC
	HR	*p*-value		HR	*p*-value	
SSS	1.08 (1.05–1.11)	<0.0001*	871.5	1.07 (1.04–1.10)	<0.0001*	877.3
SRS	1.09 (1.05–1.13)	<0.0001*	877.1	1.08 (1.04–1.12)	0.0002*	879.5
SDS	1.11 (1.05–1.18)	0.0003*	880.1	1.09 (1.02–1.16	0.0129*	885.9
Stress LVEF	0.97 (0.95–0.98)	<0.0001*	873.6	0.97 (0.95–0.98)	<0.0001*	871.0
Rest LVEF	0.97 (0.95–0.98)	<0.0001*	872.4	0.96 (0.95–0.98)	<0.0001*	867.0
Stress EDV	1.01 (1.01–1.01)	<0.0001*	862.9	1.01 (1.01–1.01)	<0.0001*	865.0
Rest EDV	1.01 (1.01–1.01)	<0.0001*	869.1	1.01 (1.01–1.01)	<0.0001*	870.8
Stress ESV	1.01 (1.01–1.01)	<0.0001*	863.3	1.01 (1.01–1.01)	<0.0001*	863.2
Rest ESV	1.01 (1.01–1.01)	<0.0001*	865.7	1.01 (1.01–1.01)	<0.0001*	866.3
Difference stress LVEF − rest LVEF^a^	1.72 (0.07–43.08)	0.7427	890.9	5.71 (0.19–167.74)	0.3125*	890.0
Difference stress EDV − rest EDV^a^	1.03 (1.01–1.04)	0.0003*	878.7	1.03 (1.01–1.04)	0.0001*	878.4
Difference stress ESV − rest ESV^a^	1.03 (1.01–1.05)	0.0093*	884.5	1.02 (1.01–1.04)	0.005*	884.0

SPECT, single-photon emission computed tomography; AIC, Akaike information criterion; SRS, summed rest score; SDS, summed difference score; SSS, summed stress score; LVEF, left ventricular ejection fraction; EDV, end-diastolic volume; ESV, end-systolic volume.

* Significant *p*-values < 0.05.

^a^ Difference values refer to the absolute difference between stress and rest measurements.

### 3.6. Mortality prediction by LVEF subgroup

Although ESV outperformed LVEF in predicting all-cause mortality, LVEF is a practical and widely used prognostic indicator. Table 5 shows the hazard ratios for mortality for stress and rest LVEF divided into three groups: LVEF >50% (reference group); LVEF 35%–50%; and LVEF <35%. For example, the hazard ratio for upright stress LVEF <35% compared to LVEF >50% was 3.00 (95% CI: 1.68–5.34), and for LVEF 35%–50% it was 1.95 (95% CI: 1.18–3.25). Hazard ratios showed similar trends for upright rest, supine stress, and supine rest LVEF.

**Table 5:** Mortality hazard ratios by LVEF >50%, 35–50%, and <35%.

		Upright		Supine	
	vs. >50%	HR	*p*-value	HR	*p*-value
Stress LVEF			0.0005*		0.0002*
	<35%	3.00 (1.68–5.34)		3.24 (1.81–5.79)	
	35–50%	1.95 (1.18–3.25)		1.85 (1.12–3.08)	
Rest LVEF			<0.0001*		<0.0001*
	<35%	3.70 (2.16–6.34)		3.67 (2.05–6.58)	
	35–50%	1.42 (0.83–2.44)		2.32 (1.40–3.85)	

HR, hazard ratio; LVEF, left ventricular ejection fraction.

* Significant *p*-values < 0.05.

### 3.7. Multivariable analysis for mortality prediction

**Figure 2:**
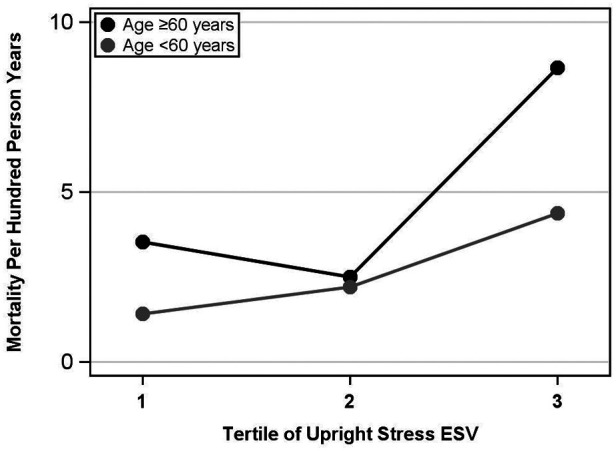
Upright stress ESV tertiles vs. mortality, ages <60 years and ages ≥60 years. Upright stress ESV is divided into tertiles, ordered from lowest to highest ESV, vs. mortality events per hundred person-years. The gray curve represents patients aged <60 and the black curve represents ages ≥60. The overall mortality rate was significantly higher in the third tertile compared with the second, but not significantly different between the first and second tertiles. ESV, end-systolic volume.

Multivariable modeling for all-cause mortality demonstrated that upright stress ESV, combined with age, diabetes, and cardiovascular disease status, was the best predictor of all-cause mortality with the lowest AIC. The best model for the supine position included the same demographic variables in addition to supine resting ESV. The partial likelihood ratio test showed the equivalent predictive ability of these two best models. See Figure 2 for a graphical representation of the mortality rate per 100 person-years by upright stress ESV tertile.

### 3.8. Indexed volumes sensitivity analysis

A sensitivity analysis was performed using volumetric indices corrected for body surface area for the ESV and EDV areas. The indices were included in the multivariable prediction models but did not provide additional predictive ability and were therefore removed from the final analyses.

## 4. Discussion

This study demonstrates that upright and supine D-SPECT data provide the equivalent predictive ability for identifying angiographic CAD and risk for all-cause mortality. In multivariable models that include commonly available demographic variables, the addition of left ventricular volume measurements provides a similar prediction of all-cause mortality, whether acquired in the supine or upright position.

This study helps address the question of the optimal imaging position for volumetric measurements on the D-SPECT camera. It has previously been suggested that volumetric measurements are best obtained in the supine position for adequate comparison with other cardiac imaging modalities (33). Nevertheless, a validation study comparing upright CZT volumes on the D-SPECT camera with cardiac magnetic resonance volumes showed a good correlation for LVEF, although upright CZT images underestimated supine ESV and EDV (31). This underestimation probably reflects reduced left ventricular filling in the upright position. The present study does not compare the accuracy of upright vs. supine measurements of LVEF with a reference standard such as cardiac magnetic resonance. It does suggest, however, that the prognostic values of upright and supine volumes are similar. It also shows that, while LVEF and EDV are statistically smaller in the upright position, the absolute differences are likely clinically insignificant (a 1% difference in LVEF).

Obese patients in the present study had a higher mortality rate than non-obese patients. It has been previously observed that obese patients have higher end-diastolic volumes than non-obese patients (34). In the present study, we also noted that end-diastolic volumes were statistically higher in the supine position compared to the upright position, but the change in end-diastolic volumes by imaging position was minimal. The observed differences in upright vs. supine end-diastolic volumes may be more closely related to left ventricular loading conditions than to body weight.

An important additional implication from this study is that ESV is a stronger predictor of all-cause mortality than LVEF, regardless of whether it is measured in the supine or upright position. A possible physiological explanation is that ESV is a proxy for systolic dysfunction and remodeling. Numerous previous studies have supported the strength of ESV as a predictor of events (17, 19, 35–39), although not all studies have demonstrated the strength of ESV over LVEF for prediction (40).

The final major finding of this study was that SSS is the strongest predictor of angiographic CAD, whether performed in the upright or supine position. This finding is in contrast to a previous, smaller sample that showed improved prediction in the supine position (6). This, however, does not detract from the value of imaging in multiple projections to avoid confounding attenuation artifacts with true perfusion defects.

There are some potential limitations to the present study. This was a retrospective review of patients at a single urban academic medical center. Our patients had a high mortality rate of nearly 20% over a median follow-up of 75 months. We suspect that this is due to the complex multimorbidity of our patient population and thus may not be easily generalizable. We measured all-cause mortality rather than cardiovascular death, which may capture unrelated mortality events. We did not correlate upright and supine volumetric measurements with another imaging modality, such as cardiac magnetic resonance, but other generally applied imaging modalities are not available for upright imaging. Finally, the administered radioactivity at the time of the present study was higher than the substantially lower doses used with the CZT camera in our current practice, but this is not expected to alter the present findings.

In conclusion, in the present study, angiographic CAD was best predicted by the combination of supine SSS and LVEF, although this supine model was not statistically superior to the next-best upright model. The study demonstrates that all-cause mortality was best predicted by ESV, in combination with age, DM status, and cardiovascular disease (CVD) status, with equivalent predictive ability in both the upright and supine positions. Finally, it shows that LVEF and EDV measurements are statistically different in the upright compared to the supine position, but their absolute differences are clinically insignificant.

## Data Availability

The raw data supporting the conclusions of this article will be made available by the authors, without undue reservation.

## References

[1] Oldan JD Shaw LK Hofmann P Phelan M Nelson J Pagnanelli R Prognostic value of the cadmium-zinc-telluride camera: a comparison with a conventional (Anger) camera. J Nucl Cardiol. (2016) 23(6):1280–7. 10.1007/s12350-015-0181-9 26122879

[2] Nudi F Iskandrian AE Schillaci O Peruzzi M Frati G Biondi-Zoccai G. Diagnostic accuracy of myocardial perfusion imaging with CZT technology: systemic review and meta-analysis of comparison with invasive coronary angiography. JACC Cardiovasc Imaging. (2017) 10(7):787–94. 10.1016/j.jcmg.2016.10.023 28330657

[3] Bailliez A Lairez O Merlin C Piriou N Legallois D Blaire T Left ventricular function assessment using 2 different cadmium-zinc-telluride cameras compared with a *γ*-camera with cardiofocal collimators: dynamic cardiac phantom study and clinical validation. J Nucl Med. (2016) 57(9):1370–5. 10.2967/jnumed.115.168575 27127220

[4] Gimelli A Bottai M Quaranta A Giorgetti A Genovesi D Marzullo P. Gender differences in the evaluation of coronary artery disease with a cadmium-zinc telluride camera. Eur J Nucl Med Mol Imaging. (2013) 40(10):1542–8. 10.1007/s00259-013-2449-0 23703458

[5] Abbott BG Case JA Dorbala S Einstein AJ Galt JR Pagnanelli R Contemporary cardiac SPECT imaging—innovations and best practices: an information statement from the American Society of Nuclear Cardiology. Circ Cardiovasc Imaging. (2018) 11(9):e000020. 10.1161/HCI.0000000000000020 30354679

[6] Athar MW Waqar F Dwivedi AK Ahmad S Sanghvi S Scott E Effects of gender and defect reversibility on detection of coronary disease with an upright and supine cadmium-zinc-telluride camera. J Nucl Cardiol. (2021) 28(4):1569–82. 10.1007/s12350-019-01878-7 31489586

[7] Engbers EM Timmer JR Mouden M Knollema S Jager PL Ottervanger JP. Prognostic value of myocardial perfusion imaging with a cadmium-zinc-telluride SPECT camera in patients suspected of having coronary artery disease. J Nucl Med. (2017) 58(9):1459–63. 10.2967/jnumed.116.188516 28450561

[8] Lima R Peclat T Soares T Ferreira C Souza AC Camargo G. Comparison of the prognostic value of myocardial perfusion imaging using a CZT-SPECT camera with a conventional anger camera. J Nucl Cardiol. (2017) 24(1):245–51. 10.1007/s12350-016-0618-9 27510176

[9] Miller RJH Han D Rozanski A Gransar H Friedman JD Hayes S CZT camera systems may provide better risk stratification for low-risk patients. J Nucl Cardiol. (2021) 28(6):2927–36. 10.1007/s12350-020-02128-x 32500175

[10] Jameria ZA Abdallah M Fernandez-Ulloa M O’Donnell R Dwivedi AK Washburn E Analysis of stress-only imaging, comparing upright and supine CZT camera acquisition to conventional gamma camera images with and without attenuation correction, with coronary angiography as a reference. J Nucl Cardiol. (2018) 25(2):540–9. 10.1007/s12350-017-0781-7 28108979

[11] Ben-Haim S Almukhailed O Neill J Slomka P Allie R Shiti D Clinical value of supine and upright myocardial perfusion imaging in obese patients using the D-SPECT camera. J Nucl Cardiol. (2014) 21(3):478–85. 10.1007/s12350-014-9853-0 24477404

[12] Chawla D Rahaby M Amin AP Vashistha R Alyousef T Martinez HX Soft tissue attenuation patterns in stress myocardial perfusion SPECT images: a comparison between supine and upright acquisition systems. J Nucl Cardiol. (2011) 18(2):281–90. 10.1007/s12350-010-9336-x 21234826

[13] Bednárová V Kincl V Kamínek M Vašina J Panovský R Máchal J. The prognostic value of ultra low-dose thallium myocardial perfusion protocol using CZT SPECT. Int J Cardiovasc Imaging. (2019) 35(6):1163–7. 10.1007/s10554-019-01535-7 30680654

[14] Nakazato R Tamarappoo BK Kang X Wolak A Kite F Hayes SW Quantitative upright-supine high-speed SPECT myocardial perfusion imaging for detection of coronary artery disease: correlation with invasive coronary angiography. J Nucl Med. (2010) 51(11):1724–31. 10.2967/jnumed.110.078782 20956478 PMC3130607

[15] Bourque J Velazquez E Tuttle R Shaw L Oconnor C Borgesneto S. Mortality risk associated with ejection fraction differs across resting nuclear perfusion findings. J Nucl Cardiol. (2007) 14(2):165–73. 10.1016/j.nuclcard.2006.11.011 17386378

[16] Al Saikhan L Park C Tillin T Mayet J Chaturvedi N Hughes A. 3 3d echocardiography-derived indices of left ventricular function and structure predict long-term mortality differently in men and women: the Southall and Brent revisited (SABRE) study. Heart (2019) 10:A3–5. Available at: https://heart.bmj.com/lookup/doi/10.1136/heartjnl-2019-BCS.3 (Accessed February 15, 2022). 10.1136/heartjnl-2019-BCS.3

[17] Sharir T Germano G Kavanagh PB Lai S Cohen I Lewin HC Incremental prognostic value of post-stress left ventricular ejection fraction and volume by gated myocardial perfusion single photon emission computed tomography. Circulation. (1999) 100(10):1035–42. 10.1161/01.CIR.100.10.1035 10477527

[18] Wu D Zhang Z Ma R Guo F Wang L Fang W. Comparison of CZT SPECT and conventional SPECT for assessment of contractile function, mechanical synchrony and myocardial scar in patients with heart failure. J Nucl Cardiol. (2019) 26(2):443–52. 10.1007/s12350-017-0952-6 28623525

[19] McManus DD Shah SJ Fabi MR Rosen A Whooley MA Schiller NB. Prognostic value of left ventricular end-systolic volume index as a predictor of heart failure hospitalization in stable coronary artery disease: data from the heart and soul study. J Am Soc Echocardiogr. (2009) 22(2):190–7. 10.1016/j.echo.2008.11.005 19084372 PMC2675872

[20] Kattoor AJ Kolkailah AA Iskander F Iskander M Diep L Khan R The prognostic value of regadenoson SPECT myocardial perfusion imaging: the largest cohort to date. J Nucl Cardiol. (2021) 28(6):2799–807. 10.1007/s12350-020-02135-y 32383079

[21] Cochet H Bullier E Gerbaud E Durieux M Godbert Y Lederlin M Absolute quantification of left ventricular global and regional function at nuclear MPI using ultrafast CZT SPECT: initial validation versus cardiac MR. J Nucl Med. (2013) 54(4):556–63. 10.2967/jnumed.112.110577 23385955

[22] Cherk MH Ky J Yap KSK Campbell P McGrath C Bailey M Optimal reproducibility of gated sestamibi and thallium myocardial perfusion study left ventricular ejection fractions obtained on a solid-state CZT cardiac camera requires operator input. J Nucl Cardiol. (2012) 19(4):713–8. 10.1007/s12350-012-9561-6 22547397

[23] Sharir T Slomka PJ Hayes SW DiCarli MF Ziffer JA Martin WH Multicenter trial of high-speed versus conventional single-photon emission computed tomography imaging: quantitative results of myocardial perfusion and left ventricular function. J Am Coll Cardiol. (2010) 55(18):1965–74. 10.1016/j.jacc.2010.01.028 20430269

[24] Kracskó B Barna S Sántha O Kiss A Varga J Forgács A Effect of patient positioning on the evaluation of myocardial perfusion SPECT. J Nucl Cardiol. (2018) 25(5):1645–54. 10.1007/s12350-017-0865-4 28361477

[25] Nakaya K Onoguchi M Nishimura Y Kiso K Otsuka H Nouno Y Comparison between prone and upright imaging of the Inferior wall using 201TlCl myocardial perfusion SPECT. J Nucl Med Technol. (2017) 45(4):304–8. 10.2967/jnmt.117.197632 29042470

[26] Schaefer WM Lipke CSA Kühl HP Koch KC Kaiser HJ Reinartz P Prone versus supine patient positioning during gated 99mTc-sestamibi SPECT: effect on left ventricular volumes, ejection fraction, and heart rate. J Nucl Med. (2004) 45(12):2016–20. 15585475

[27] Hain SF Van Gramberg D Bomanji JB Kayani I Groves AM Ben-Haim S. Can upright myocardial perfusion imaging be used alone with a solid-state dedicated cardiac camera? Q J Nucl Med Mol Imaging. (2013) 57(4):383–90. 23752688

[28] Doukky R Rahaby M Chawla D Vashistha R Alyousef T Amin AP. Soft tissue attenuation patterns associated with upright acquisition SPECT myocardial perfusion imaging: a descriptive study. Open Cardiovasc Med J. (2012) 6(1):22–7. 10.2174/1874192401206010022 22435079 PMC3308262

[29] Maddahi J Mendez R Mahmarian JJ Thomas G Babla H Bai C Prospective multicenter evaluation of rapid, gated SPECT myocardial perfusion upright imaging. J Nucl Cardiol. (2009) 16(3):351–7. 10.1007/s12350-009-9063-3 19247734

[30] Manyari DE Kostuk WJ. Left and right ventricular function at rest and during bicycle exercise in the supine and sitting positions in normal subjects and patients with coronary artery disease. Assessment by radionuclide ventriculography. Am J Cardiol. (1983) 51(1):36–42. 10.1016/S0002-9149(83)80008-3 6849265

[31] Coupez E Merlin C Tuyisenge V Sarry L Pereira B Lusson JR Validation of cadmium–zinc–telluride camera for measurement of left ventricular systolic performance. J Nucl Cardiol. (2018) 25(3):1029–36. 10.1007/s12350-017-0816-0 28194726

[32] Poliner LR Dehmer GJ Lewis SE Parkey RW Blomqvist CG Willerson JT. Left ventricular performance in normal subjects: a comparison of the responses to exercise in the upright and supine positions. Circulation. (1980) 62(3):528–34. 10.1161/01.CIR.62.3.528 7398013

[33] Daquarti G Meretta A Masoli O. Importance of patient positioning in left ventricular function assessment [letter to the editor]. J Nucl Cardiol. (2019) 26(3):1019. 10.1007/s12350-017-1004-y 28755082

[34] Dorbala S Crugnale S Yang D Di Carli MF. Effect of body mass index on left ventricular cavity size and ejection fraction. Am J Cardiol. (2006) 97(5):725–9. 10.1016/j.amjcard.2005.09.122 16490446

[35] Kwon DH Menon V Houghtaling P Lieber E Brunken RC Cerqueira MD Predictive value of exercise myocardial perfusion imaging in the Medicare population: the impact of the ability to exercise. Cardiovasc Diagn Ther. (2014) 4(1):5–12. 10.3978/j.issn.2223-3652.2014.02.09 24649419 PMC3943782

[36] Kato M Kitada S Kawada Y Nakasuka K Kikuchi S Seo Y Left ventricular end-systolic volume is a reliable predictor of new-onset heart failure with preserved left ventricular ejection fraction. Cardiol Res Pract. (2020) 2020:e3106012. 10.1155/2020/3106012 PMC7341373 32670635

[37] White HD Norris RM Brown MA Brandt PW Whitlock RM Wild CJ. Left ventricular end-systolic volume as the major determinant of survival after recovery from myocardial infarction. Circulation. (1987) 76(1):44–51. 10.1161/01.CIR.76.1.44 3594774

[38] Turakhia MP McManus DD Whooley MA Schiller NB. Increase in end-systolic volume after exercise independently predicts mortality in patients with coronary heart disease: data from the heart and soul study. Eur Heart J. (2009) 30(20):2478–84. 10.1093/eurheartj/ehp270 19578167 PMC2761597

[39] Upton MT Rerych SK Newman GE Port S Cobb FR Jones RH. Detecting abnormalities in left ventricular function during exercise before angina and ST-segment depression. Circulation. (1980) 62(2):341–9. 10.1161/01.CIR.62.2.341 7397975

[40] Burns RJ Gibbons RJ Yi Q Roberts RS Miller TD Schaer GL The relationships of left ventricular ejection fraction, end-systolic volume index and infarct size to six-month mortality after hospital discharge following myocardial infarction treated by thrombolysis. J Am Coll Cardiol. (2002) 39(1):30–6. 10.1016/S0735-1097(01)01711-9 11755283

